# Theory of mechanochemical patterning in biphasic biological tissues

**DOI:** 10.1073/pnas.1813255116

**Published:** 2019-02-28

**Authors:** Pierre Recho, Adrien Hallou, Edouard Hannezo

**Affiliations:** ^a^University Grenoble Alpes, CNRS, Laboratoire Interdisciplinaire de Physique, F-38000 Grenoble, France;; ^b^Cavendish Laboratory, Department of Physics, University of Cambridge, Cambridge, CB3 0HE, United Kingdom;; ^c^Wellcome Trust/Cancer Research UK Gurdon Institute, University of Cambridge, Cambridge, CB2 1QN, United Kingdom;; ^d^Wellcome Trust/Medical Research Council Stem Cell Institute, University of Cambridge, Cambridge, CB2 1QR, United Kingdom;; ^e^Institute of Science and Technology Austria, 3400 Klosterneuburg, Austria

**Keywords:** morphogenesis, pattern formation, morphogen transport, poroelasticity, scaling

## Abstract

The formation of self-organized patterns is key to the morphogenesis of multicellular organisms, although a comprehensive theory of biological pattern formation is still lacking. Here, we propose a minimal model combining tissue mechanics with morphogen turnover and transport to explore routes to patterning. Our active description couples morphogen reaction and diffusion, which impact cell differentiation and tissue mechanics, to a two-phase poroelastic rheology, where one tissue phase consists of a poroelastic cell network and the other one of a permeating extracellular fluid, which provides a feedback by actively transporting morphogens. While this model encompasses previous theories approximating tissues to inert monophasic media, such as Turing’s reaction–diffusion model, it overcomes some of their key limitations permitting pattern formation via any two-species biochemical kinetics due to mechanically induced cross-diffusion flows. Moreover, we describe a qualitatively different advection-driven Keller–Segel instability which allows for the formation of patterns with a single morphogen and whose fundamental mode pattern robustly scales with tissue size. We discuss the potential relevance of these findings for tissue morphogenesis.

## Significance

Pattern formation is a central question in developmental biology. Alan Turing proposed that this could be achieved by a diffusion-driven instability in a monophasic system consisting of two reacting chemicals. In this paper, we extend Turing’s work to a more realistic mechanochemical model of multicellular tissue, modeling also its biphasic and mechanical properties. Overcoming limitations of conventional reaction–diffusion models, we show that mechanochemical couplings between morphogen concentrations and extracellular fluid flows provide alternative, non-Turing, mechanisms by which tissues can form robust spatial patterns.

How symmetry is broken in the early embryo to give rise to a complex organism is a central question in developmental biology. To address this question, Alan Turing proposed an elegant mathematical model where two reactants can spontaneously form periodic spatial patterns through an instability driven by their difference in diffusivity (1). Molecular evidence of such a reaction–diffusion scheme in vivo remained long elusive, until pairs of activator–inhibitor morphogens were proposed to be responsible for pattern formation in various embryonic tissues (2–9). Interestingly, these studies also highlight some theoretical and practical limitations of existing reaction–diffusion models, including the fact that Turing patterns require the inhibitor to diffuse at least one order of magnitude faster than the activator ($D_{I}/D_{A}>10$) (3), although most morphogens are small proteins of similar molecular weights, implying that $D_{I}/D_{A}\approx 1$. As a consequence, the formation of Turing patterns in vivo should result from other properties of the system such as selective morphogen immobilization (10–12) or active transport (13) as demonstrated in synthetic systems. Moreover, reaction–diffusion models of pattern formation entail a number of restrictions regarding the number and interactions of morphogens and pattern scaling with respect to the tissue size, which have been all limiting their quantitative applicability in vivo. While the genetic and biochemical aspects of developmental pattern formation have been the focus of most investigations, the interplay between mechanics and biochemical processes in morphogenesis started to unfold following some pioneering contributions (14). The crucial role played by multiphasic tissue organization and active cell behaviors in biological pattern formation is now an active field of research (15–18).

In this article, we derive a general mathematical formulation of tissues as active biphasic media coupled with reaction–diffusion processes, where morphogen turnover inside cells, import/export at the cell membrane, and active mechanical transport in the extracellular fluid are coupled together through tissue mechanics. While encompassing classical reaction–diffusion results (1–4), for instance allowing import–export mechanisms to rescale diffusion coefficients and to form patterns with equally diffusing morphogens (11), this theory provides multiple routes to robust pattern formation. In particular, assuming a generic coupling between intracellular morphogen concentration and poroelastic tissue mechanics, we demonstrate the existence of two fundamentally different non-Turing patterning instabilities, respectively assisted and driven by advective extracellular fluid flows, explaining pattern formation with only a single morphogen with robust scaling properties and how patterning can be independent of underlying morphogen reaction schemes. Finally, we discuss the biological relevance of such a model and in particular its detailed predictions that could be verified in vivo.

## Results

### Derivation of the Model.

**Fig. 1.**
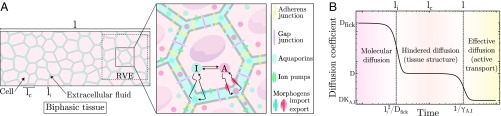
Model for pattern formation in active biphasic tissues. (*A*) Schematic of the model. (*Left*) Cells form a poroelastic network, permeated by extracellular fluid, where three natural length scales can be defined: the interstitial space size ($l_{i}$), the characteristic cell size ($l_{c}$), and the tissue size (l). (*Right*) Biochemical interactions between morphogens, A and I, take place inside the cell and are described by their respective turnover rate functions f(A,I) and g(A,I). A and I are exported across the cell membrane at rates $\lambda_{A,I}$ and imported at rates $\gamma_{A,I}$, respectively. In the extracellular space, both A and I spread freely by diffusion at the same rate D or can be advected by the fluid at velocity $v_{e}$. (*B*) Evolution of the effective diffusion coefficient as a function of time and space scales. At shorter distances and times, diffusive behavior of morphogens is described by a molecular diffusion coefficient, $D_{\text{Fick}}$. At intermediate scales, the diffusive motion of morphogens starts to be hindered by cells and the global diffusion coefficient, D, depends of the tissue spatial organization through $\phi^{*}$. At larger scales, morphogen diffusion is controlled by dynamic interactions with cells (import/export, adsorption/desorption,) and an effective coefficient $DK_{A,I}$ (9).

As sketched in Fig. 1*A*, we model multicellular tissues as continuum biphasic porous media of typical length l, with a first phase consisting of a poroelastic network made of adhesive cells of arbitrary shape and typical size $l_{c}$ (with local volume fraction ϕ) and a second phase of aqueous extracellular fluid permeating in between cells in gaps of a characteristic size $l_{i}$. These two internal length scales disappear in the coarse-graining averaging over a representative volume element of typical length scale $l_{r}$ satisfying $l_{i,c}\ll l_{r}\ll l$. Both phases are separated by cell membranes, actively regulating the interfacial exchange of water and other molecules due to genetically controlled transport mechanisms (19, 20). At the boundary of the domain, no-flux boundary conditions are imposed such that the system is considered in isolation. We present below the main steps of the model derivation, which are detailed in *SI Appendix*.

#### Intracellular morphogen dynamics.

Morphogens enable cell–cell communication across the tissue and determine cell fate decisions. Importantly, most known morphogens cannot directly react together and, as such, have to interact “through” cells (or cell membranes) where they are produced and degraded (20). Concentration fields of two morphogens, $A_{i,e}\left(\vec{r},t\right)$ and $I_{i,e}\left(\vec{r},t\right)$, are thus defined separately in each phase of the system, with indexes (i,e) denoting intra- and extracellular phases, respectively. The conservation laws of the intracellular phase, which cannot be transported, read

$$\begin{array}{c} \partial_{t}\left(\phi A_{i}\right)=f\left(A_{i},I_{i}\right)+\gamma_{A}A_{e}-\lambda_{A}A_{i}\\ \partial_{t}\left(\phi I_{i}\right)=g\left(A_{i},I_{i}\right)+\gamma_{I}I_{e}-\lambda_{I}I_{i}, \end{array}$$ [1]

where $\partial_{t}$ denotes the partial derivative with respect to time and $\gamma_{A,I}$ (resp. $\lambda_{A,I}$) the import (resp. export) rates of morphogens (which can also describe immobilization rates at the cell membrane). We also introduce f and g, the nonlinear morphogen turnover rates describing their production and degradation by cells, with a single stable equilibrium solution $f\left(A_{i}^{*},I_{i}^{*}\right)=g\left(A_{i}^{*},I_{i}^{*}\right)=0$. Finally, we introduce the transmembrane transport equilibrium constants by $K_{A}=\lambda_{A}/\gamma_{A}$ and $K_{I}=\lambda_{I}/\gamma_{I}$. Although the import/export coefficients $K_{A,I}$ could in principle depend on morphogen concentrations, this constitutes a nonlinear effect that we ignore in our linear theory.

#### Extracellular fluid dynamics.

Next, we write a mass conservation equation for the incompressible fluid contained in the tissue interstitial space between cells,

$$\begin{array}{c} \partial_{t}\phi -\nabla \cdot \left(\left(1-\phi \right)v_{e}\right)=\frac{\phi_{h}\left(A_{i},I_{i}\right)-\phi }{\tau }, \end{array}$$ [2]

where $v_{e}$ is the velocity of the extracellular fluid. The right-hand side of this equation describes the fact that cells actively regulate their relative volume fraction to a homeostatic value $\phi_{h}\left(A_{i},I_{i}\right)$ at a time scale τ (21). Note that Eq. **2** with $v_{e}\neq \;0$ implies a recirculation of internal fluid, via gap junctions (22) (*SI Appendix*, section 1.A.3).

As detailed below, we assume that local cellular morphogen concentrations have an influence on the volume fraction ϕ which couples tissue mechanics to local morphogens concentration in our theory. At linear order, this coupling generically reads $\phi_{h}\left(A_{i},I_{i}\right)=\phi^{*}\;+\;\chi_{A}\left(A_{i}-A_{i}^{*}\right)/A_{i}^{*}\;+\;\chi_{I}\left(I_{i}-I_{i}^{*}\right)/I_{i}^{*}$, where we denote $\phi^{*}=\phi_{h}\left(A_{i}^{*},I_{i}^{*}\right)$ the equilibrium cell volume fraction, and the $\chi_{A,I}$ terms account for the sensitivity of cell volume to intracellular morphogen concentrations. Such a mechanochemical effect on the tissue packing fraction, ϕ, can occur either via the active control of individual cell volume (21) or through the active balance between cell proliferation and loss (*SI Appendix*, section 1.A.4), with $\chi_{A,I}>0$ for morphogens acting as growth factors and $\chi_{A,I}<0$ for morphogens working as growth inhibitors. This is a reasonable assumption, as a number of morphogens involved in cell fate decisions can act as growth factor/inhibitors (23, 24), and in vitro experiments have shown that cells, upon exposure to factors such as FGF or EGF, elicit a series of signaling-mediated responses involving an increase in transmembrane ion flux, cell volume changes (21), and subsequent cell growth/division (25). Moreover, during digits pattern formation in the limb bud, which has been proposed to rely on a Turing instability, morphogens such as BMP participate in both the reaction–diffusion scheme (8) and morphogenetic events such as cell condensation (26), with skeletal formation being associated with large cell volume fraction changes (27). The cell volume fraction is thus highly modulated in space and time, concomitantly with morphogen pattern formation (26), advocating for the need of a global mechanochemical theory taking into account both effects.

#### Extracellular morphogen dynamics.

Morphogens, once secreted by cells, are transported by diffusion and advection in the extracellular fluid,

$$\begin{array}{c} \partial_{t}\left(\left(1-\phi \right)A_{e}\right)+\nabla \cdot \left(\left(1-\phi \right)A_{e}v_{e}-D\nabla A_{e}\right)\;=\;-\gamma_{A}A_{e}+\lambda_{A}A_{i}\\ \partial_{t}\left(\left(1-\phi \right)I_{e}\right)+\nabla \cdot \left(\left(1-\phi \right)I_{e}v_{e}-D\nabla I_{e}\right)\;=\;-\gamma_{I}I_{e}+\lambda_{I}I_{i}, \end{array}$$ [3]

where D is the global Fickian diffusion coefficient of both morphogens, depending on tissue packing and tortuosity (9, 28, 29). As we are interested in a linear theory, we consider here $D=D\left(\phi^{*}\right)$ as a constant. We neglect here, for the sake of simplicity, phenomena such as extracellular morphogen degradation or the influence of extracellular morphogen concentrations on reaction terms, as they do not modify qualitatively the dynamics (*SI Appendix*, section 1.C). Note that one could also take into account, at the mesoscopic level, some effective nonlocal interactions such as cell–cell communication via long-ranged cellular protrusions (30). This may require one to consider spatial terms in Eq. **1** to introduce an additional characteristic length scale from nonlocal cell–cell transport.

#### Mechanical behavior of the cellular phase.

To complete our description, we need to specify a relation linking cell volume fraction to interstitial fluid velocity. For this, we use a poroelastic framework, whose applicability to describe the mechanical response of biological tissues has been thoroughly investigated in various contexts (31, 32). Taking a homogeneous tissue as a reference state, poroelastic properties imply that a local change of the cell volume fraction creates elastic stresses in the cellular phase which translate to gradients of extracellular fluid pressure p. Such gradients of pressure in turn drive extracellular fluid flows, which can advect morphogens, and we show (*SI Appendix*, section 1.A.7) that this effects results in a simple Darcy’s law between cell volume fraction and fluid flow (29):

$$\begin{array}{c} \left(1-\phi \right)v_{e}=-\frac{\kappa }{\eta }\nabla p=D_{m}\nabla \phi . \end{array}$$ [4]

This relation introduces the hydrodynamic diffusion coefficient of the extracellular fluid, $D_{m}=K\kappa /\eta$, a key mechanical parameter of the model which feeds back on the reaction–diffusion dynamics in Eq. **3**, with κ the tissue permeability, K the elastic drained bulk modulus, and η the fluid viscosity. The hydrodynamic length scale $l_{m}=\sqrt{D_{m}\tau }$ is associated to such fluid movement. Importantly, we explore here only the simplest tissue rheology for the sake of simplicity and concision. Nevertheless, we also investigate (*SI Appendix*, section 1.H) the role of growth and plastic cell rearrangements and show that they can be readily incorporated in our model, leading to different types of patterning instabilities. However, we highlight here that the results presented thereafter are all robust to small to intermediate levels of tissue rearrangements.

### Model of an Active Biphasic Tissue.

Eqs. **1**–**4** define a full set of equations describing the chemo-mechanical behavior of an active biphasic multicellular tissue (*SI Appendix*, section 1.B). To provide clear insights on the biophysical behavior of the system, we focus on a limit case where $\gamma_{A,I}\gg \lambda_{A,I}\gg f,g$ such that $K_{A,I}\ll 1$. This corresponds to an ubiquitous biological situation where rates of membrane transport are orders of magnitude faster than transcriptionally controlled morphogen turnover rates and where endocytosis occurs at a much faster rate than exocytosis. In that case, the relations $A_{e}≃K_{A}A_{i}$ and $I_{e}≃K_{I}I_{i}$ always hold and even if a significant fraction of morphogens is immobilized inside the cells (9), the import/export terms cannot be neglected as $\gamma_{A,I}$ are very large, so that $\gamma_{A}\left(A_{e}-K_{A}A_{i}\right)$ and $\gamma_{I}\left(I_{e}-K_{I}I_{i}\right)$ are indeterminate quantities (*SI Appendix*, section 1.C). Summing both internal Eq. **1** and external Eq. **3** conservation laws, we obtain a simplified description of the system (*SI Appendix*, section 1.C):

$$\begin{array}{c} \partial_{t}\left(\phi A_{i}\right)+\nabla \cdot \left(A_{i}K_{A}D_{m}\nabla \phi -K_{A}D\nabla A_{i}\right)\;=\;f\left(A_{i},I_{i}\right)\\ \partial_{t}\left(\phi I_{i}\right)+\nabla \cdot \left(I_{i}K_{I}D_{m}\nabla \phi -K_{I}D\nabla I_{i}\right)\;=\;g\left(A_{i},I_{i}\right)\\ -l_{m}^{2}\Delta \phi +\phi =\phi_{h}\left(A_{i},I_{i}\right). \end{array}$$ [5]

Nondimensionalizing times with $\tau_{A}$ associated with the degradation of $A_{i}$ in the morphogen turnover functions f and g and lengths with $l_{A}=\sqrt{K_{A}D\tau_{A}}$, we find that Eq. **5** is controlled by a few nondimensional parameters: $K_{I}/K_{A}$ describes the mismatch of morphogen membrane transport, $D_{m}/D$ compares the global hydrodynamic and Fickian diffusion of the morphogens, $\tau /\left(K_{A}\tau_{A}\right)$ compares the response time of cell volume fraction to the effective morphogen turnover rate, and $\chi_{A}$ and $\chi_{I}$ account for the sensitivity of ϕ to morphogen levels. Using this restricted set of parameters encapsulating the behavior of the model, we investigate several of its biologically relevant limits, demonstrating that they provide independent routes toward tissue patterning.

### Orders of Magnitude on Morphogen Transport.

In the simplest limit of the model, the cell fraction remains constant, $\phi =\phi^{*}$, which is valid if the effect of the morphogens on ϕ is very small compared with the restoring mechanical forces (i.e., $\chi_{A,I}=0$). The model then reduces to Turing’s original system, with diffusion coefficients being renormalized by morphogen transmembrane transport equilibrium constants, $K_{A,I}D$, similar to results obtained in refs. 9 and 11. This implies that even species with similar D can exhibit effective diffusion coefficients widely differing from each other on longer time scales and produce Turing patterns when $K_{I}\gg K_{A}$ (*SI Appendix*, section 1.F).

In Fig. 1*B*, we depict scaling arguments for the changes in effective diffusion coefficient at various time/length scales, associated with both tissue structure and import/export kinetics (11). At small time scales, diffusion is characterized by a local Fickian diffusion coefficient, theoretically expected to be of the order of $D_{\text{Fick}}\approx 10^{-11}{\text{m}}^{2}\cdot {\text{s}}^{-1}$, in line with fluorescence correlation spectroscopy (FCS) measurements (7, 9, 20). This occurs across a typical cell-to-cell distance of $l_{i}\approx 10^{-7}-10^{-9}$ m (33), so that this regime is valid for time scales below $l_{i}^{2}/D_{\text{Fick}}\approx 10^{-2}-10^{-6}$ s, which is much faster than the typical import/export kinetics of $1/\gamma_{A,I}\approx 10^{1}-10^{2}$ s (34). At intermediate timescales, the diffusion coefficient needs to be corrected for volume exclusion effects due to the porous nature of the tissue, an effect which can be very large for cell volume fraction close to one (35). An upper bound (Hashin–Shtrikman) for global diffusion can be computed, irrespective of the microscopic details of tissue geometry, as $D\left(\phi^{*}\right)\leq D_{\text{Fick}}\left(1-\phi^{*}\right)/\left(1+\phi^{*}/2\right)$ (28), which would suggest, in the case of $\phi^{*}\approx 0.8-0.9$, that it should be around an order of magnitude smaller than local diffusion, $D\left(\phi^{*}\right)\approx 10^{-12}{\text{m}}^{2}\cdot {\text{s}}^{-1}$. Finally, at the time scales larger than $1/\gamma_{A,I}$ described by the present model, the diffusion is decreased further by a factor $K_{A,I}$, i.e., by the relative concentrations of morphogens “trapped” cellularly (i.e., a 1:10 ratio) such that $D\left(\phi^{*}\right)K_{A,I}\approx 10^{-12}-10^{-13}{\text{m}}^{2}\cdot {\text{s}}^{-1}$. This is consistent with effective diffusion coefficients measured from tissue-wide fluorescence recovery after photobleaching (FRAP) over minutes to hours time scales (7, 9, 20, 35). Note here that the respective contributions of volume exclusion and import/export effects on FRAP-measured diffusion coefficients are nontrivial and are detailed in *SI Appendix*, section 1.H.

Overall, although our model in its simplest limit ($\phi =\phi^{*}$) relaxes the classical Turing condition $D_{I}\gg D_{A}$, it still implies quite stringent conditions on the ratio of intracellular and extracellular morphogens ($I_{e}/I_{i}\gg A_{e}/A_{i}$). Exploring further the effect of a variable cell volume fraction ϕ, we demonstrate that coupling morphogen dynamics and tissue mechanics suppresses this limitation via active transport of morphogens.

### Turing–Keller–Segel Instabilities.

To assess the regions in parameter space where stable patterns can form in our mechanochemical framework, we perform a linear stability analysis on Eq. **5**. Here, we consider a classical Gierer–Meinhardt activator–inhibitor scheme (2), $f\left(A,I\right)=\rho A^{2}/I-A/\tau_{A}$ and $g\left(A,I\right)=\rho A^{2}-I/\tau_{I}$, where ρ is the rate of activation and inhibition and $\tau_{A,I}$ are the time scales of degradation of A and I (2) and the particular case of a single morphogen capable of increasing $\phi_{h}$ ($\chi_{A}>0,\chi_{I}=0$).

**Fig. 2.**
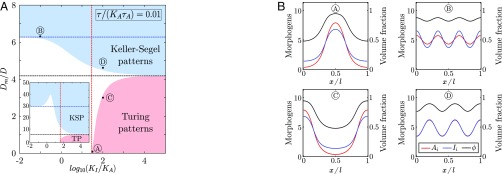
Linear stability analysis and numerical simulations of pattern formation in active biphasic tissues. (*A*) Phase diagram of Eq. **5** in the $\left(K_{I}/K_{A},D_{m}/D\right)$ parameter space for $\tau /\left(K_{A}\tau_{A}\right)=0.01$ and $\tau /\left(K_{A}\tau_{A}\right)=0.1$ (*Inset*). The red and blue dashed lines correspond to analytical thresholds of instability (given in the text) for Turing and Keller–Segel patterns, respectively. The black dashed line is the analytical phase boundary between both regimes in the limit $K_{I}\gg K_{A}$ given by $\chi_{A}=D/D_{m}+\tau /\left(\tau_{A}K_{A}\right)$. This limit is shifted up when the ratio $\tau /\tau_{A}K_{A}$ is increased, while a pronounced notch appears in the “Keller–Segel patterns” domain (*Inset*). Other parameters are set to $\chi_{A}=0.25$, $\chi_{I}=0$, $\tau_{I}/\left(K_{A}\tau_{A}\right)=0.2$, $K_{A}\tau_{A}\rho =1$, $\phi^{*}=0.85$, and large tissue size ($l_{A}/l\ll 1$). (*B*) One-dimensional numerical simulations of Eq. **5** with random initial conditions for several choices of parameters identified by letters A, B, C, and D. $l_{A}/l=0.1$.

**Fig. 3.**
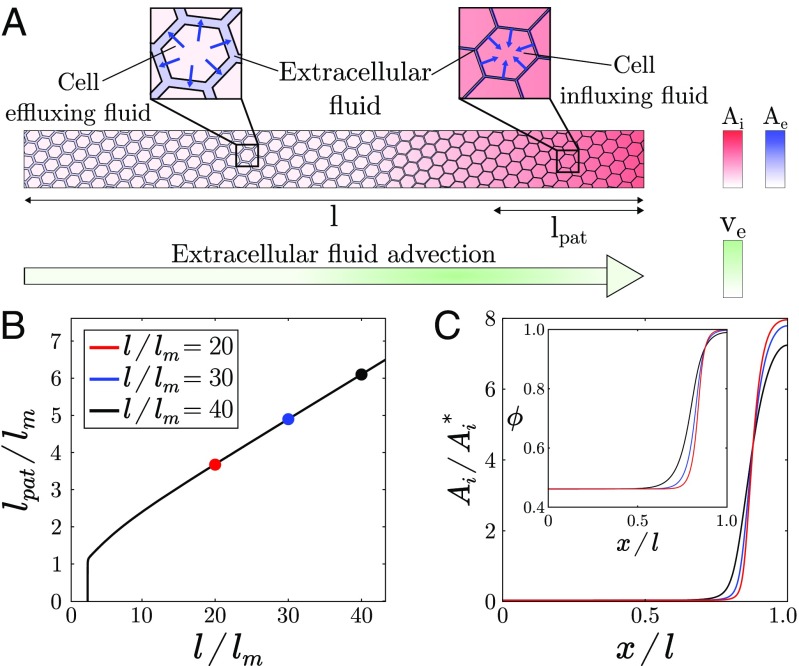
Scaling properties of the Keller–Segel instability with one morphogen. (*A*) Schematic of the Keller–Segel instability in a 1D tissue. Morphogen gradients generate cell volume fraction gradients (via local fluid exchanges, blue arrows in *Inset*), which in return cause mechanically induced self-amplifying extracellular flows that advect morphogens from morphogen-poor to morphogen-rich regions (green arrow). (*B*) Normalized pattern size as a function of system size in the single-morphogen case with f=0. (*C*) Morphogen concentration and cell packing fraction (*Inset*) profiles remain quasi-stationary as system size increases. Parameters are $\chi_{A}=0.25$, $D_{m}/D=10$, and $\phi^{*}=0.85$.

In the phase diagram in Fig. 2*A*, we show that two distinct instabilities can be captured by this simplified theory. The first instability, identified here as “Turing patterns,” corresponds to a classical Turing instability, where diffusive transport of morphogens dominates over their advection by interstitial fluid ($D_{m}\ll D$) and with instability threshold given by $K_{I}\tau_{I}-K_{A}\tau_{A}>2\sqrt{\tau_{A}\tau_{I}K_{A}K_{I}}$ for $l_{A}/l\ll 1$(dashed red line in Fig. 2*A*) which, as expected, is always true regardless of the value of $\tau_{A,I}$ if $K_{I}\gg K_{A}$. However, another generic pattern forming instability driven by active transport phenomena is present in the phase diagram, labeled “Keller–Segel patterns” in Fig. 2*A* (36). The physical origin of the resulting pattern is here similar to active fluid instabilities (15, 17, 37–40): If stochastic local changes in morphogen concentration result in an increase in cell volume fraction, fluid must be pumped inside cells. This causes local elastic deformations in the tissue which generate large-scale extracellular fluid flows from regions of low to high morphogen concentration, resulting in a positive feedback loop of morphogen enrichment (Fig. 3*A*) and steady-state patterns. Interestingly, such an instability can occur even for a single morphogen. In this limit, patterning occurs if $\sqrt{\chi_{A}}>\sqrt{D/D_{m}}+\sqrt{\tau /\left(\tau_{A}K_{A}\right)}$ when $l_{A}/l\ll 1$ so that the volume fraction sensitivity $\chi_{A}$ is above a critical value (dashed blue line in Fig. 2*A*, which captures well the phase boundary in the limit $K_{A}\gg K_{I}$, although the instability occurs generically for any value of $K_{A,I}$). The number of patterns displayed by the profiles shown in Fig. 2*B* can be predicted by linear analysis (*SI Appendix*, section 1.D) because they are chosen close to the onset of instability.

Thus, coupling tissue mechanical behavior to morphogen reaction–diffusion provides, via the generation of advective fluid flows, a route to stable pattern formation with a single morphogen. Moreover, this instability has two remarkable features. First, it requires only the presence of a single morphogen (*SI Appendix*, section 1.G) which could correspond to many practical situations where an activator/inhibitor pair has not been clearly identified, for instance the role of Wnt in the antero-posterior pattern of planarians (41). Second, it possesses spatial scaling properties regarding to its fundamental mode, compared with a Turing instability. Indeed, when morphogen turnover rate is small compared with its effective hydrodynamic and Fickian diffusion (f→0), the fundamental mode, i.e., a single two-zones pattern, is the most unstable in a robust manner, given that morphogen turnover f stabilizes specifically this mode (*SI Appendix*, section 1.G.2), whereas in the case of a Turing instability, this would require fine-tuning and marginally stable reaction kinetics. We illustrate such a scaling property in Fig. 3. This mechanism could potentially apply to situations where a binary spatial pattern is independent of system size such as dorso-ventral or left–right patterns in early vertebrate embryos (7, 9) or planarian antero-posterior patterns (41, 42). If so, it could provide a simpler alternative to previously proposed mechanisms involving additional species or complex biochemical signaling pathways (7, 42).

Importantly, simple estimates can be used to demonstrate the biological plausibility of such mechanical effects during morphogenetic patterning. A key parameter driving Keller–Segel instabilities is the hydrodynamic diffusion coefficient $D_{m}$, which can be estimated from values of the drained bulk modulus $K\approx 10^{4}$ Pa (31) and the tissue permeability upper bound (28) $\kappa \approx l_{i}^{2}\left(1-\phi^{*}\right)/\left(1+\phi^{*}/2\right)$ with $l_{i}\approx 10^{-7}-10^{-9}$ m and $\phi^{*}\approx 0.85$ as above. Using $\eta \approx 10^{-3}$ Pa⋅s (water viscosity), we obtain $D_{m}\approx 10^{-12}-10^{-8}{\text{m}}^{2}\cdot {\text{s}}^{-1}$, showing that the hydrodynamic diffusion can be similar to or even much larger than Fickian diffusion. In agreement with typical time scales involved in regulatory volume increase or decrease of cells following an osmotic perturbation (21), we estimate that $\tau \approx 10^{2}$ s, while the morphogen turnover time scale has been measured as $\tau_{A}\approx 10^{4}-10^{5}$ s (9). With $K_{A}\approx 0.1$ as above, we obtain $\tau /\left(K_{A}\tau_{A}\right)\approx 0.01-0.1$, which is used in Fig. 2 and displays broad regions of instability, although parameters like sensitivities $\chi_{A,I}$ would need to be better assessed in vivo in future works.

### Cross-Diffusion Turing Instabilities.

Finally, we investigate the behavior of our model (Eq. **5**), when cell fraction sensitivity to morphogen concentration is negative ($\chi_{A,I}<0$), eliminating the possibility of uphill morphogen diffusion at the origin of the Keller–Segel instability. We also consider that f and g do not necessarily follow activator–inhibitor kinetics, but any possible interaction scheme between two morphogens. For mathematical clarity on the physical nature of the instability studied here, we make the simplifying assumptions that τ=0 and $\chi_{A,I}\ll 1$, with $D∼D_{m}\chi_{A,I}$ in Eq. **5**. This relates to a realistic biological situation, where cell volume fraction relaxes rapidly after perturbation and depends weakly on morphogen levels, yielding

$$\begin{array}{c} \phi^{*}\partial_{t}A_{i}+\nabla \cdot \left(A_{i}K_{A}D_{m}\nabla \phi_{h}-K_{A}D\nabla A_{i}\right)\;=\;f\left(A_{i},I_{i}\right)\\ \phi^{*}\partial_{t}I_{i}+\nabla \cdot \left(I_{i}K_{I}D_{m}\nabla \phi_{h}-K_{I}D\nabla I_{i}\right)\;=\;g\left(A_{i},I_{i}\right). \end{array}$$ [6]

In this limit, the conditions for linear stability of the homogeneous solution are exactly the ones of a classical Turing system but with cross-diffusion terms (*SI Appendix*, section 1.E). Such a scenario has been studied in the framework of monophasic reaction–diffusion systems with ad hoc cross-diffusion terms (43), which arise generically in various chemical and biological systems (44). Our work thus provides a particular biophysical interpretation of these terms in multicellular tissues, which we show to originate from intrinsically mechanochemical feedbacks between morphogen dynamics and tissue mechanics.

**Fig. 4.**
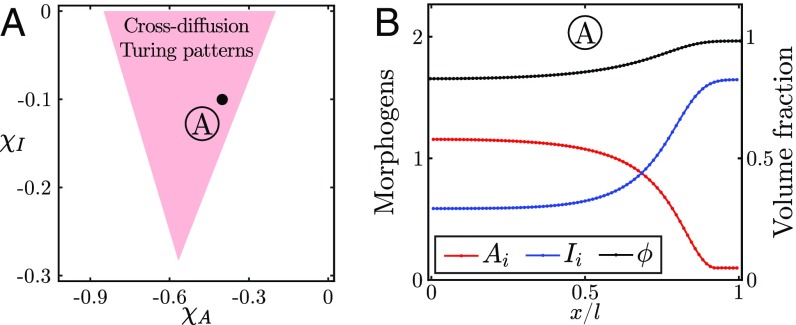
Pattern formation for cross-diffusion Turing instabilities. (*A*) Phase diagram of Eq. **5** in the $\left(\chi_{A},\chi_{I}\right)$ space obtained by numerical linear stability analysis. Parameters are $\tau /\left(K_{A}\tau_{A}\right)=0.01$, $D_{m}/D=10$, $K_{I}/K_{A}=10$, $\tau_{I}/\left(K_{A}\tau_{A}\right)=0.9$, $\phi^{*}=0.85$, and $l_{A}/l\ll 1$. (*B*) One-dimensional numerical simulation of Eq. **5** using a simple inhibitor–inhibitor reaction scheme (*SI Appendix*, section 1.B).

As shown in ref. 43, such cross-diffusion terms result in a dramatic broadening of the phase space for patterns. In particular, any two-morphogen reaction scheme can now generate spatial patterns and not just the classical activator–inhibitor schemes. For instance, it becomes possible to obtain patterns with activator–activator or inhibitor–inhibitor kinetics similar to those observed in numerous gene regulatory networks or signaling pathways involved in cell fate decisions (45). We illustrate this result by considering an inhibitor–inhibitor kinetic scheme, which cannot yield patterns in the classical Turing framework, and demonstrate analytically and numerically the existence of a region of stable patterns (from Eq. **5**), where a cross-diffusion–driven Turing instability can develop (Fig. 4).

## Discussion

In this paper, we have introduced a generalization of Turing’s work on pattern formation in biological tissues by coupling equations describing the structure and mechanical properties of multicellular tissues with a classical reaction–diffusion scheme. In particular, our work highlights two important features of multicellular tissues, as of yet largely unexplored in this context: their biphasic nature, i.e., the fact that morphogen production/degradation is controlled by cells while transport takes place extracellularly requiring active membrane exchanges [effectively rescaling diffusion (9, 11)], and the possibility for active large-scale flows to develop within the tissue interstitial space. We demonstrate that coupling tissue cell volume fraction to local morphogen levels [based on the dual role of morphogens in patterning and cell growth/volume regulation (23, 24)] provides a biophysically realistic route toward two qualitatively different modes of patterning instability. Extracellular fluid flows can have two important consequences on patterning. First, as the Turing instability is rooted in the cross-effects between a stable chemical reaction of two morphogens and their diffusion, the conditions of such instability are deeply affected by active hydrodynamic transport which can create cross terms in the effective diffusion matrix. This causes a drastic widening of the phase space of Turing patterning, rendering it robust and only weakly dependent on the morphogen reaction scheme. Second, extracellular fluid flows can also create an instability of a different nature (Keller–Segel), when these flows have an antidiffusive structure, spontaneously creating morphogen gradients. Here, chemical reactions between morphogens are setting only the number of patterns, and if such reactions are sufficiently slow, the spatial pattern of the morphogen always coarsens to the fundamental mode of instability and has robust scaling properties compared with conventional Turing models. This could have interesting implications concerning recent experimental evidence for robust scaling of the Nodal/Lefty pattern in the early zebrafish embryo (46).

In this respect, our approach, which has the advantage of parsimony, taking into account the manifest biphasic nature of multicellular tissues, is complementary to others which have been proposed to solve limitations of Turing’s model by introducing additional morphogen regulators (42, 47) and also displays connections with recent developments in the mechanochemical descriptions of active fluids such as the cell cytoskeleton (15, 16). Nevertheless, although our hypothesis of cell volume fraction gradients driving large-scale flows is generic to biphasic tissues, further quantitative experiments would be needed to test the relationship between morphogen concentration and cell volume fraction, as well as probe the role of transmembrane import/export kinetics or similar phenomena such as transmembrane signaling (11), morphogen adsorption/desorption on the cell surface (9), and long-distance cellular protrusions (30), on effective morphogen diffusion rates. Systems such as digits patterning, where the cell volume fraction spatial pattern appears concomitant to morphogen patterns (26), or planarian antero-posterior patterning, where activator/inhibitor pairs have not been clearly identified (41), provide possible testing grounds for our model. Interestingly, large-scale extracellular fluid flows have been increasingly observed during embryo development, not only in the classical case of cilia-driven flows (48), but also due to mechanical forces arising from cellular contractions as well as osmotic and poro-viscous effects (49, 50), calling for a more systematic understanding of passive vs. active transport mechanisms during embryonic pattern formation. Whether biological examples of Turing patterning instabilities, such as left–right or dorso-ventral patterning, digits pattern formation, or skin appendage patterns, are causally associated with concomitant changes in cell volume and/or cell packing remains a result to be experimentally investigated.

## Methods

Linear stability analysis was performed numerically using Mathematica, while numerical integrations of the model equations were performed using a custom-made Matlab code.

## Supplementary Material

Supplementary File
